# New insights for the regulatory feedback loop between type 1 crustacean female sex hormone (*CFSH-1*) and insulin-like androgenic gland hormone (*IAG*) in the Chinese mitten crab (*Eriocheir sinensis*)

**DOI:** 10.3389/fphys.2022.1054773

**Published:** 2022-10-31

**Authors:** Dandan Zhu, Tianyi Feng, Nan Mo, Rui Han, Wentao Lu, Shucheng Shao, Zhaoxia Cui

**Affiliations:** ^1^ School of Marine Sciences, Ningbo University, Ningbo, China; ^2^ Laboratory for Marine Biology and Biotechnology, Qingdao National Laboratory for Marine Science and Technology, Qingdao, China

**Keywords:** crustacean female sex hormone, insulin-like androgenic gland hormone, intersex crab, early embryogenesis, secondary sexual characteristic, sexual sex differentiation

## Abstract

To clarify the hormone control on sex determination and differentiation, we studied the Chinese mitten crab, *Eriocheir sinensis* (Henri Milne Edwards, 1854), a species with importantly economic and ecological significance. The crustacean female sex hormone (CFSH) and the insulin-like androgenic gland hormone (IAG) have been found to be related to the sex determination and/or differentiation. *CFSH-1* of *E. sinensis* (*EsCFSH-1*) encoded a 227 amino-acid protein including a signal peptide, a CFSH-precursor-related peptide, and a mature CFSH peptide. Normally, *EsCFSH-1* was highly expressed in the eyestalk ganglion of adult female crabs, while the expression was declined in the intersex crabs (genetic females). The intersex crabs had the androgenic glands, and the expression level of *EsIAG* was close to that of male crabs. During the embryogenesis and larval development, the changes of *EsCFSH-1* and *EsIAG* genes expression in male and female individuals were shown after the zoea IV stage. Next, we confirmed the existence of the regulatory feedback loop between *EsCFSH-1* and *EsIAG* by RNA interference experiment. The feminization function of *EsCFSH-1* was further verified by examining the morphological change of external reproductive organs after *EsCFSH-1* knockdown. The findings of this study reveal that the regulatory interplay between CFSH and IAG might play a pivotal role in the process of sex determination and/or differentiation in decapod crustaceans.

## 1 Introduction

The crustacean female sex hormone (CFSH), initially identified in the blue crab, *Callinectes sapidus*, was a neuropeptide hormone synthesized in the X-organ and secreted by the sinus gland in the eyestalk (Zmora and Chung, 2014). Self-explained by its name, the function of CFSH was found to be related to the female sexual sex differentiation of crustaceans, especially the development of the female reproductive anatomies such as gonopores and ovigerous setae (Adkins-Regan, 2014) and possibly the ovarian maturation (Liu et al., 2018; Tsutsui et al., 2018). To date, *CFSH* has been identified in several crustaceans such as *Carcinus maenas*, *Sagmariasus verreauxi*, *Cherax quadricarinatus*, *Procambarus clarkii*, *Chorismus antarcticus*, *Penaeus japonicus*, *Penaeus monodon*, *Scylla paramamosain*, *Macrobrachium rosenbergii*, and *Lysmata vittata* (Farhadi et al., 2021; Toyota et al., 2021). Nonetheless, the relevant information was lacking in *Eriocheir sinensis*, an economically and ecologically important crustacean.

Discovered in 2007, the insulin-like androgenic gland hormone (IAG) secreted from the androgenic gland (AG) has been proven as a critical peptide hormone regulating male differentiation in decapod species. The masculinizing function of IAG was described as an “IAG-switch” in the endocrine regulatory axis of X-organ-sinus-gland (XO-SG)-androgenic gland (AG)-testis (Levy et al., 2020; Levy and Sagi, 2020). Studies have found that repressing *IAG* expression at early developmental stages could induce the degeneration of primary and secondary sex characteristics of male, which could eventually lead to feminization (Ventura et al., 2012; Levy et al., 2017; Priyadarshi et al., 2017). To date, IAG orthologs have been identified in several crustacean species, including *Eriocheir sinensis* (Song et al., 2018; Fu et al., 2020; Cui et al., 2021).

*CFSH* has been regarded as an upstream regulator of *IAG*, while the recent evidence supported the possible existence of a negative regulatory loop between *CFSH* and *IAG*. Previous studies found that *CFSH* of *Scylla paramamosain* could inhibit the expression of *IAG in vitro* (Liu et al., 2018) and the inhibitory mechanism was later detailed explained (Jiang et al., 2020a; Jiang et al., 2020c), while *IAG1* and *CFSH* of *Lysmata vittata* was able to inhibit the expression of each other reciprocally (Liu et al., 2021b; Liu et al., 2021c). However, the research of the hormone control on sex determination and differentiation has been limited for lacking of sex identification during the embryogenesis and larval development.

In this study, based on the genomic and transcriptomic information, the basic gene feature of *CFSH-1* was analyzed in *E. sinensis*. Furthermore, we examined the possibility of existing crosstalk between *CFSH-1* and *IAG* by comparing their expression level at various developmental stages including distinguished sex embryos and larvae, normal and intersex crabs. At last, we confirmed the regulatory feedback loop by observing the change of gene expression after knocking down its potential inhibitor. The new insights based on the results from embryogenesis and larval individuals and intersex crabs in our study will hopefully contribute to a better understanding of the process of sex determination and differentiation in the Chinese mitten crab and other crustaceans alike.

## 2 Methods and materials

### 2.1 Animals and samples

The adults, intersex crabs, larvae and embryos of *E. sinensis* were collected from the local crab farms and hatcheries around Xinghua, Jiangsu province of China. The developmental stages of embryos and larvae were classified according to the previous descriptions (Liang et al., 1974; Montú et al., 1996) under an Olympus BX53F2 stereo microscope (Olympus, Tokyo, Japan). The biological samples were separated in tubes, and rapidly frozen in liquid nitrogen until further usage.

Animal handling and experimental procedures were performed according to the guidelines of the Guide for the Use of Experimental Animals of Ningbo University.

### 2.2 Total RNA extraction and cDNA synthesis for tissue samples

Total RNA was extracted from tissues including eyestalk ganglion (EG), ovary (O), testis (T), androgenic gland (AG) using the TRIZOL^®^ reagent (Invitrogen, California, United States) according to the manufacturer’s instructions. The concentration and quality of total RNA were assessed using a NanoDrop One spectrophotometer (Thermo Fisher Scientific, Massachusetts, United States). 1 µg of total RNA was converted to cDNA using the PrimeScript™ RT reagent Kit with gDNA Eraser (TaKaRa, Tokyo, Japan) following the manufacturer’s instructions.

### 2.3 DNA/RNA co-extraction for embryos and larvae

The DNA/RNA co-extraction was applied for the single embryo at the stages of fertilized egg (Fe), two-cell (2C), and blastula (Bs) and the single larva at the stage of zoea III (Z3), zoea IV (Z4), and zoea V (Z5). Total DNA and RNA of each embryo and larva were extracted simultaneously using a Tiangen DP423 DNA/RNA/Protein Isolation Kit (Tiangen, Beijing, China). Firstly, a specimen was fully homogenized in the lysis buffer by a Servicebio KZ-III-F low-temperature tissue grinder (Servicebio Technology, Wuhan, China). After leaving on ice for 15 min, the total DNA was separated by centrifuging the lysate through a HiBind^®^ DNA mini-column at 13,000 × g for 1 min at room temperature using a Sorvall^™^ Legend^™^ Micro 17R microcentrifuge (Thermo Fisher Scientific, Waltham, US). After mixing with absolute ethanol and later DNA digestion by DNase I for 15 min, the total RNA in the flow-through lysate was collected by centrifugation through a HiBind^®^ RNA mini-column at 13,000 × g for 1 min. Subsequently, the total DNA and RNA were eluted into separated tubes by centrifuging the wash buffer through the columns twice (13,000 × g, 1 min). Finally, the purified nucleic acids were dissolved in 100 µL DEPC-treated water. The concentration and quality of the DNA and total RNA were assessed using a NanoDrop 2000 spectrophotometer (Thermo Fisher Scientific, Massachusetts, United States). The total RNA was later reversed transcribed to cDNA by CellAmp^™^ Whole Transcriptome Amplification Kit (TaKaRa, Kyoto, Japan).

### 2.4 Distinguishing sex by genetic markers

Based on the polymerase chain reaction (PCR) method described in the published laboratory papers (Cui et al., 2018; Cui et al., 2021), the extracted genomic DNA was used to distinguish the genetic sex of *E. sinensis* with the specific primers. The total volume of PCR mixture is 25 μL, which contains 1 μL diluted cDNA template, 2.5 μL 10 × PCR buffer, 2 μL dNTP (10 mM), 0.5 μL each primer (10 mM), 0.2 μL rTaq polymerase (TaKaRa, Kyoto, Japan) and 18.3 μL double distilled H_2_O. The PCR was performed ollowing the conditions: 95°C for 3 min; 40 cycles of 95°C for 30 s, 55°C for 30 s, and 72°C for 20 s, and a final extension at 72°C for 10 min. After the agarose gel electrophoresis, the male and female can be distinguished with the specific band of about 300 base pairs (bp).

### 2.5 Cloning of the full-length cDNA

The full-length cDNA sequence of *EsCFSH-1* was extracted from the published genome assembly of *E. sinensis* (Cui et al., 2021). To verify the accuracy of the sequence, the polymerase chain reaction (PCR) was carried out using the cDNA library of female EG as the PCR template. The PCR was performed following the instruction of Ex Taq polymerase (TaKaRa, Kyoto, Japan). The total volume of PCR mixture is 25 μL, which contains 1 μL diluted cDNA template, 2.5 μL 10×PCR buffer, 2 μL dNTP (10 mM), 0.5 μL primer EsCFSH-1-F/R (10 mM), 0.3 μL Ex-Taq polymerase and 18.2 μL double distilled H_2_O. The PCR was performed following the conditions: 95°C for 3 min; 35 cycles of 95°C for 30 s, 58.5°C for 30 s, and 72°C for 40 s, and a final extension at 72°C for 10 min. The sequences of primers were listed in Table 1. The primers were designed *via* the Primer Premier 5.0 (Premier Biosoft, San Francisco, United States) and the Primer-BLAST of NCBI (ncbi.nlm.nih.gov/tools/primer-blast/) (Ye et al., 2012).

**TABLE 1:** The summary of primers used in this study.

Primer	Sequence (5′–3′)	Application
EsCFSH-1-F	CCT​TCT​CTT​GGG​ATG​TTC​G	Gene cloning
EsCFSH-1-R	ATC​TTC​ACG​GCT​TGG​GTT​C	
EsCFSH-1-qF	ATA​CGT​TGA​GCG​CCA​GAT​CC	qRT-PCR
EsCFSH-1-qR	CAG​AGC​CAC​ACA​TAC​GGA​GC	
EsIAG-qF	GCT​CCT​ACA​AGC​AGC​ACC​C	
EsIAG-qR	AGG​GTC​TTC​CAG​ATG​GAT​CG	
Es-β-actin-qF	GCA​TCC​ACG​AGA​CCA​CTT​ACA	
Es-β-actin-qR	CTC​CTG​CTT​GCT​GAT​CCA​CAT​C	
EsCFSH-1-dsF	AAC​CAC​CAT​TGT​CCA​TCC​CTC	Synthesis of dsRNA
EsCFSH-1-dsR	TTC​AGA​GCC​ACA​CAT​ACG​GAG	
EsIAG-dsF	CAC​CTC​ATG​CAA​CGT​GCA​GTT	
EsIAG-dsR	TTC​TGC​ACG​TTG​CAT​GAG​GTG	
EGFP-dsF	CAC​AAG​TTC​AGC​GTG​TCC​G	
EGFP-dsR	AAC​CAC​TAC​CTG​AGC​ACC​CA	
Primer-T7	TAA​TAC​GAC​TCA​CTA​TAG​GG	
Primer-SP6	ATTTAGGTGACACTATAG	

The PCR products separated by gel electrophoresis was excised, purified, and cloned into the pMD19-T vector (TaKaRa, Kyoto, Japan) prior to the Sanger sequencing by a commercial service provider (GENEWIZ, Suzhou, China).

### 2.6 Bioinformatics analyses

The open reading frame (ORF) was predicted by the ORF finder (https://www.ncbi.nlm.nih.gov/orffinder/) (Misener and Krawetz, 2000). The signal peptide was predicted using SignalP 5.0 Server (https://services.healthtech.dtu.dk/service.php?SignalP-5.0) (Almagro Armenteros et al., 2019). Further, the N-glycosylation motif was predicted by the NetNGlyc 1.0 Server (https://services.healthtech.dtu.dk/service.php?NetNGlyc-1.0) (Gupta and Brunak, 2002), the O-terminal glycosylation site was predicted by the NetNGlyc 1.0 Server (https://services.healthtech.dtu.dk/service.php?NetOGlyc-4.0) (Steentoft et al., 2013). The cysteine (Cys) residues and putative disulfide bonds were predicted *via* the DiANNA 1.1 web server (http://clavius.bc.edu/∼clotelab/DiANNA/) (Ferre and Clote, 2005). The secondary structure was predicted by the NovoPro software (https://www.novopro.cn/tools/secondary-structure-prediction.html) (Jones, 1999), and the SWISS-PROT software was used to analyze the tertiary spatial structure (https://swissmodel.expasy.org/) (Bairoch and Boeckmann, 1991). The conserved domain was detected by the SMART software (http://smart.embl-heidelberg.de/) (Letunic and Bork, 2018).

The multiple alignment of full-length amino acid sequences was performed by the ClustalX software using the published CFSH sequences (Table 2) excluding the signal peptide. The phylogenetic tree was constructed using the complete CFSH sequences by the MEGA6 software, in which the neighbor-joining algorithm with 2,000 bootstrap replicates was applied based on the JTT matrix-based model (Saitou and Nei, 1987).

**TABLE 2:** The source of CFSHs for the multiple sequence alignment and phylogenetic analysis.

Gene name	Species	Genebank accession number or gene ID
*SpCFSH-1*	*Scylla paramamosain*	ASU91622.1
*SpCFSH-2*		ASU91623.1
*EsCFSH-1*	*Eriocheir sinensis*	New023086.1
*EsCFSH-2a*		New 075712.1
*EsCFSH-2b*		CCG012020.1
*CmCFSH-1*	*Carcinus maenas*	AEI72264.0
*CmCFSH-2a*		GBXE01009034.1
*CmCFSH-2b*		GBXE01009035.1
*PjCFSH-ov*	*Penaeus japonicus*	BBC20727.1
*PjCFSH*		BBA53799.1
*MrCFSH-1a*	*Macrobrachium rosenbergii*	Unigene35740
*MrCFSH-1b*		Unigene35739
*MrCFSH-2a*		Unigene35630
*MrCFSH-2b*		Unigene35631
*LyvCFSH-1*	*Lysmata vittata*	QOD42430.1
*LyvCFSH-2*		QOD42431.1
*CsCFSH*	*Callinectes sapidus*	ADO00266.1
*PmCFSH*	*Penaeus monodon*	QYK79693.1
*PcCFSH-1*	*Procambarus clarkii*	GBEV01018430.1
*PcCFSH-2a*		GBEV01038769.1
*PcCFSH-2b*		GBEV01021357.1
*LivCFSH-1a*	*Litopenaeus vannamei*	JP398644.1
*LivCFSH-1b*		JP398645.1
*LivCFSH-1c*		JP398646.1

### 2.7 The preparation of short interfering double-stranded RNA

The synthesis of short interfering double-stranded RNA (dsRNA) was performed using the TranscriptAid™ T7 high yield transcription kit (Thermo Scientific Inc., Massachusetts, United States) following the standard protocols on the manual. Primers EsCFSH-1-dsF/dsR and EsIAG-dsF/dsR (Table 1) were designed to amplify cDNA fragments of *EsCFSH-1* and *EsIAG* (GenBank ID: KU724192.1) to constructed the recombined plasmids of pGEM-T-EsCFSH-1 and pGEM-T-EsIAG, respectively. Primers of EGFP-dsF/dsR (Table 1) were used to amplify the fragments of enhanced green fluorescent protein (*EGFP*) gene (GenBank: U55761) to constructed pGEM-T-EGFP. The recombined plasmids with target genes were used to get the linearized DNA templates, then the RNA transcripts can be generated from 1ug DNA templates. After extraction and purification, dsRNA could be obtained, and the concentration was measured by Nanodrop One (Thermo Scientific Inc., Massachusetts, United States) and stored at −80 °C until use.

Before the application of RNA interference, the dsRNA was diluted with the artificial crab saline (ACS: 2.570 g NaCl, 0.084g KCl, 0.148 g CaCl_2_, 0.248g MgCl_2_, 0.327 g Na_2_SO_4_, 0.238 g HEPES solved in 100 ml distilled water).

### 2.8 The gene knockdown for adult crabs

For adult crabs (body weight: 95.41 ± 10.14 g), four groups were assigned to the experiment of gene knockdown. Each group consisted of eight adult crabs (1:1 sex ratio). For the group of *IAG* or *CFSH-1* knockdown, the dsRNA of *EsIAG* or *EsCFSH-1* was injected into the hemolymph through the arthrodial membrane between the third and fourth pleopod by a 100 μL micro syringe (Shanghai Anting, Shanghai, China). For the group of blank or negative control, the equivalent amount of artificial crab saline (ACS) or dsRNA *EGFP* was injected. The quantity of delivered dsRNA was 1 µg per each gram of the body weight. Tissue samples including EG and AG of each crab were collected for RNA extraction 24 h after the RNA interference.

### 2.9 The gene knockdown for juvenile crabs

For the gene knockdown for juvenile crabs, 150 individuals at the stage of juvenile I were injected with dsRNA *EsCFSH-1* (2 μg per 1 g of body weight) using the IM11-2 and M-152 micro injection system (NARISHIGE, Tokyo, Japan). In the control group, the juveniles were injected with the equivalent amount of ACS. The samples were collected at the stage of juvenile III (around 15 days). The whole bodies of juvenile crabs were fixed in the PBS with 4% paraformaldehyde for the morphological observation.

### 2.10 The morphological observation of external reproductive structures

The gross anatomy of the male and female juveniles was observed under an Olympus SZ51 stereomicroscope (Olympus, Shinjuku, Japan). For the scanning electron microscopical observation, the samples were prepared through ethanol gradient dehydration and tert-butyl alcohol replacement. After being frozen for 20 h in -20°C, the samples were dried for 24 h in the Martin Christ Alpha 1-4LD plus freeze-dryer (Martin Christ, Osterode, Germany) before being sprayed with gold by a Hitachi E-1010 ion sputtering device (Hitachi, Tokyo, Japan). The micrographs were taken using a Hitachi S-3400N scanning electron microscope (Hitachi, Tokyo, Japan).

### 2.11 Quantitative real-time PCR

The fluorescence quantitative real-time PCR (qRT-PCR) was performed using the 7,500 Real-Time PCR system (Applied Biosystems, California, United States) with TB Green Premix Dimer Eraser (2x) (TaKaRa, Kyoto, Japan). The qRT-PCR was carried out in a total volume of 20 μL, containing 10 μL TB Green Premix Dimer Eraser (2x), 2 µL diluted cDNA, 0.5 µL forward/reverse primers (1 mM), 0.4 µL ROX Reference Dye II and 6.4 µL RNase-free water. The PCR program included the initial denaturation at 95°C for 30 s, followed by 40 cycles of denaturation at 95°C for 15 s, annealing and elongation at 60°C for 15 s and 72°C for 30 s. Each sample was run in triplicates. All primers used for the qRT-PCR were listed in Table 1. A standard curve was used to calculate the amplification efficiency and ensure primer specificity of each primer pair, EsCFSH-1-qF/R had the amplification efficiency of 105.895%, EsIAG-qF/R had the amplification efficiency of 101.054%, and the amplification efficiency of Es-β-actin-qF/R was 100.451%. The relative expression levels of target genes were calculated by the 2^−ΔΔCt^ method (Livak and Schmittgen, 2001) using *Es-β-actin* (GenBank ID: ATO74508.1) as the reference gene.

### 2.12 Statistical analysis

The normality of data was established by the Kolmogorov-Smirnov test. All the data were presented in a normal distribution and tested for variances homogeneity by the Levene’s test. The statistical analysis was performed by the one-way ANOVA followed by Tukey’s multiple tests using the GraphPad Prism seven software (GraphPad Software, San Diego, United States) (Stoline, 1981; Swift, 1997). The quantitative results were represented as mean ± SEM.

## 3 Results

### 3.1 The bioinformatics prediction of full length *EsCFSH-1* gene and protein

**FIGURE 1:**
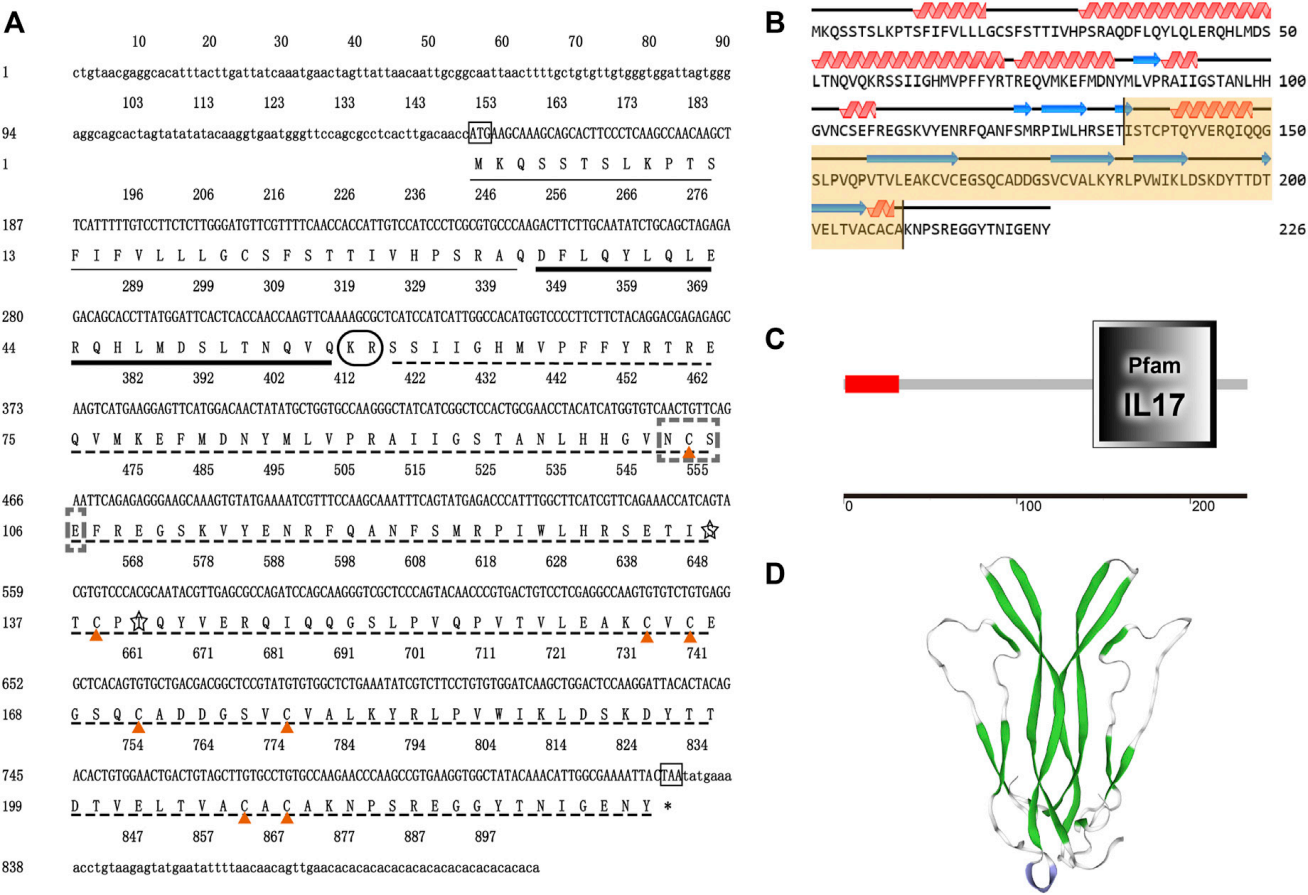
The basic molecular characterization of *EsCFSH-1* **(A)** the nucleic acids and deduced amino acids sequence of *EsCFSH-1* from the *Eriocheir sinensis*. The ORF was shown in a single letter code below the nucleotide sequence. The N-terminal glycosylation site was boxed in the dashed-line, the O-terminal glycosylation site was surrounded by the shaded stars, the cysteine residues were pointed by the orange triangles. The putative signal peptide was underlined by the thin black line, the pre-CFSH related peptide was marked by the thick black line, and the CFSH mature peptide was marked by the black dashed line **(B)** the secondary structure of EsCFSH-1 protein predicted by Novopro. The predicted interleukin-17 domain was highlighted in yellow area **(C)** the predicted protein domain by the SMART. The red box was the signal peptide, and the dark frame was the interleukin-17 domain **(D)** the tertiary protein structure of EsCFSH-1 predicted by the SWISS-PROT.

The full-length cDNA of *EsCFSH-1* (GenBank ID: OP351640) had 903 bp, included a 73-nt 3′ UTR, a 149-nt 5′ UTR, and the 681-nt ORF that encoded a protein of 226 amino acid residues (Figure 1A). The CFSH preprohormone was comprised of a signal peptide of 34 residues, a CFSH-precursor related peptide of 22 residues, a dibasic processing signal (Lys-Arg, KR), and a mature peptide of 168 residues. The molecular weight of the EsCFSH-1 protein was 25.68 kDa, and the theoretical isoelectric point was 8.06. The instability coefficient of the protein was 40.4. The average hydrophilic coefficient of the protein was -0.269. The mature peptide had a single conserved N-glycosylation site (Asn-Cys-Ser, NCS) at N103 and two O-glycosylation sites at S136 and T140. Eight cysteine residues were predicted to form four intramolecular disulfide bridges: C104-C209, C138-C171, C164-C178, and C166-C207.

The secondary structure of EsCFSH-1 contained 82 α-helixes (45.79%), 12 β-turns (5.31%), 100 random coils (44.25%), and 32 extended strands (14.16%) (Figure 1B). The domains detected by SMART showed that EsCFSH-1 had a signal peptide and an interleukin-17 (IL-17) domain (Figure 1C). The prediction of 3D structure showed the EsCFSH-1 could form a “butterfly” like homodimer (Figure 1D).

### 3.2 The phylogenetic analysis of EsCFSH-1

**FIGURE 2:**
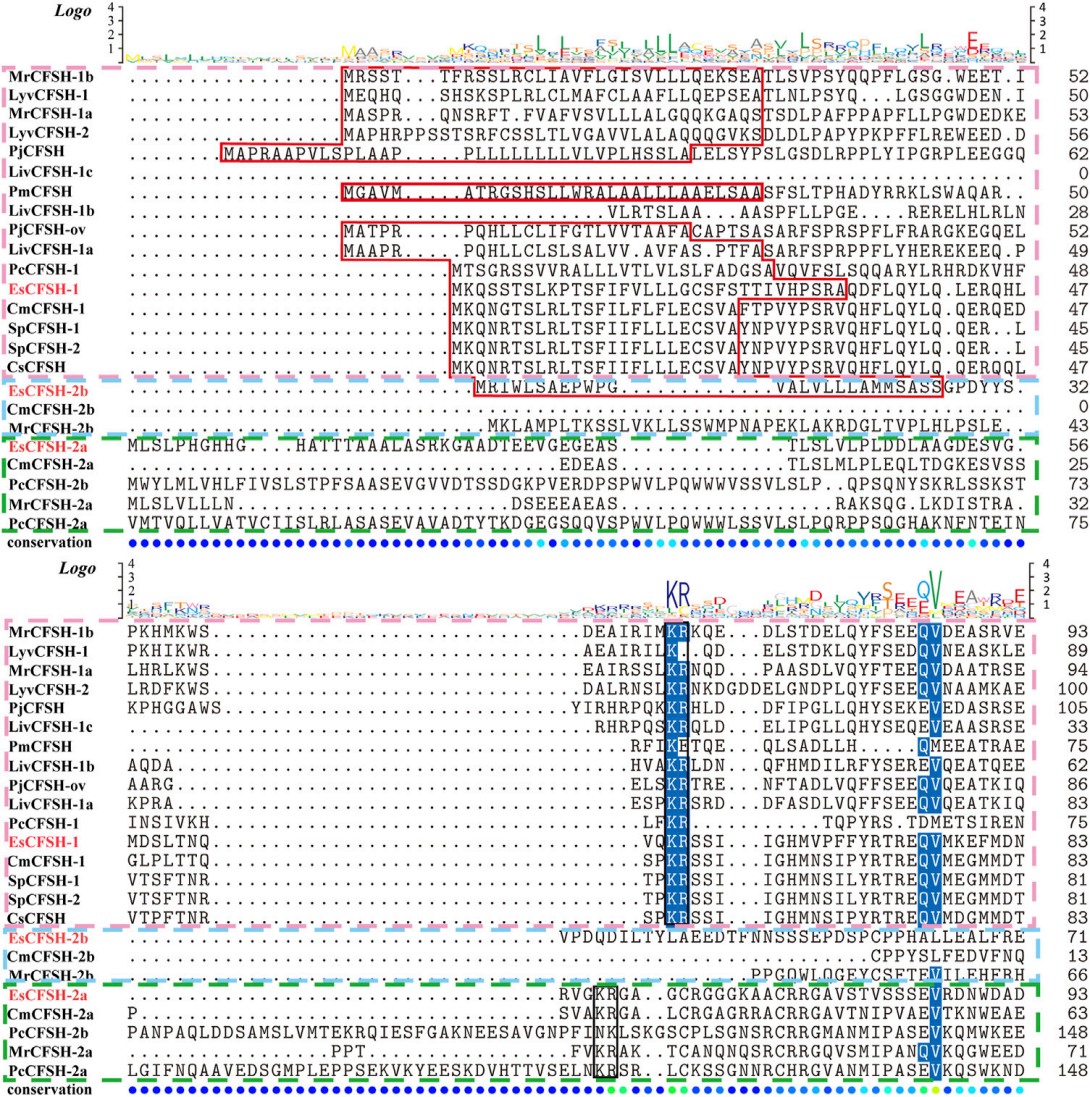
The multiple sequence alignment analysis of the amino acid of decapod CFSHs. Three subtypes of CFSHs were boxed, the pink box was CFSH 1, the light blue was CFSH 2b, and the green was CFSH 2a. The signal peptide was outlined by the red frame, and the cleavage site was boxed in black line, the conserved cysteine residues were marked with red stars.

**FIGURE 3:**
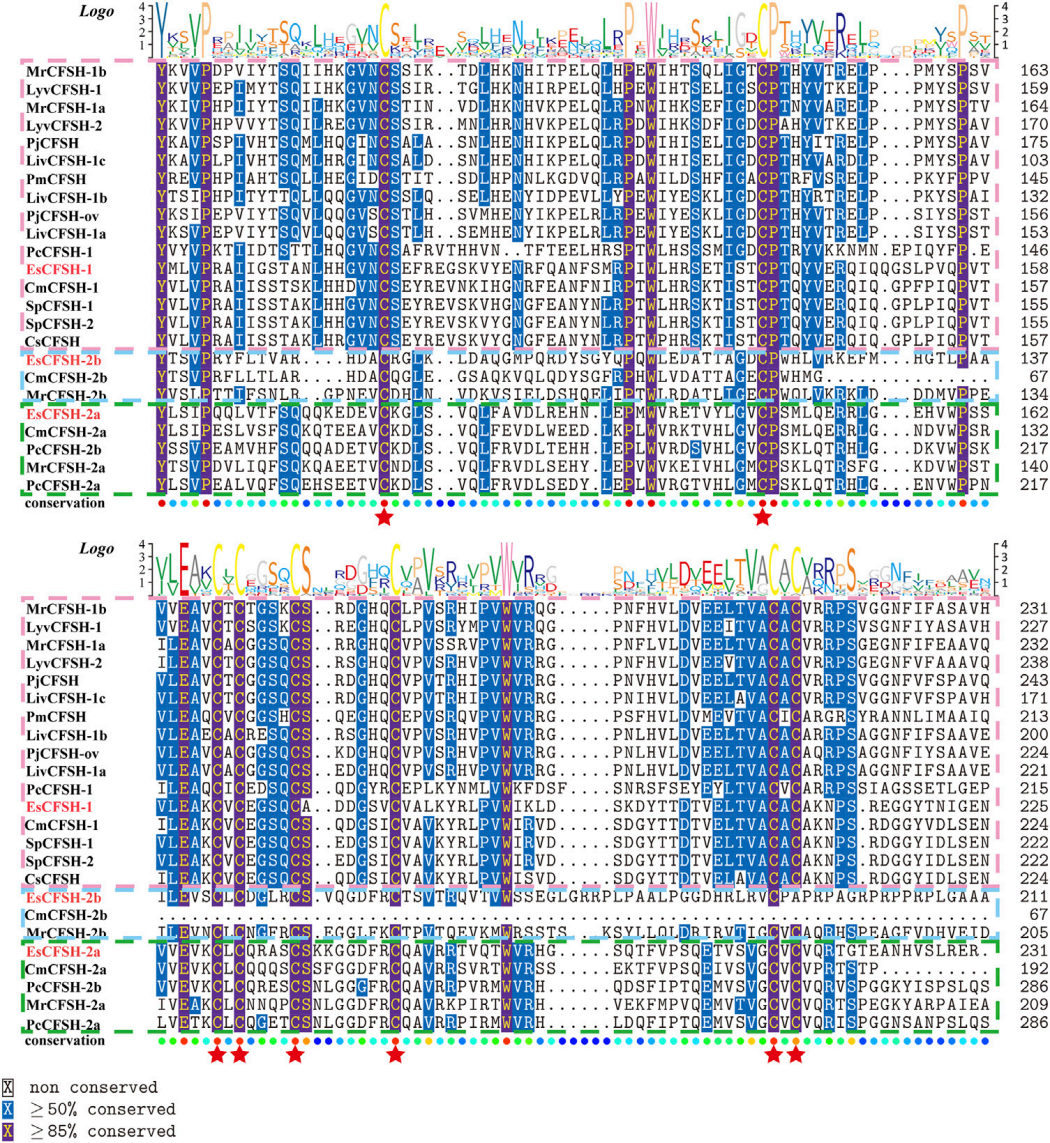
(Figure 2 continued) The multiple alignment analysis of the amino acid sequences of decapod CFSHs.

**FIGURE 4:**
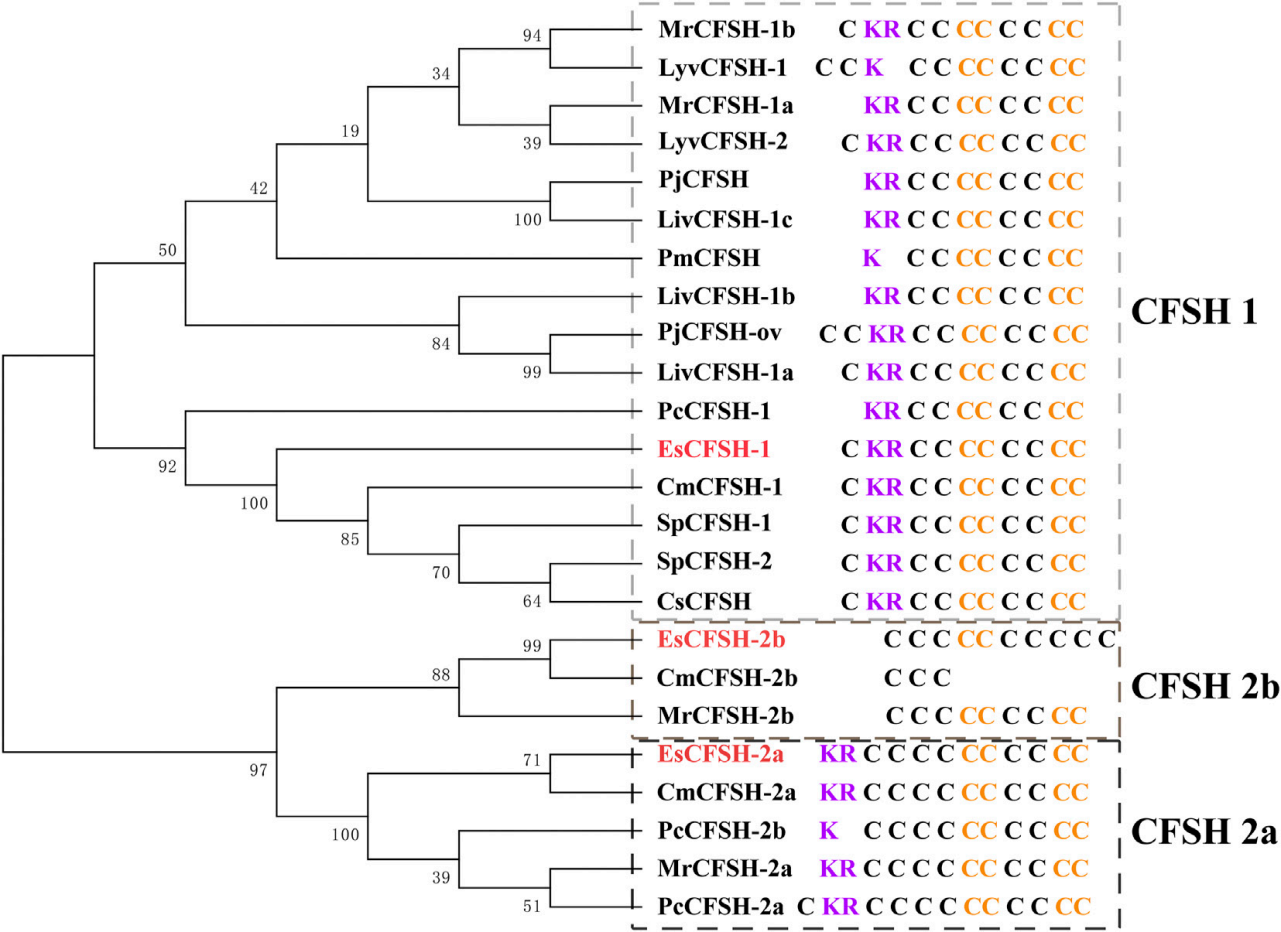
The phylogenetic analysis of the CFSH mature peptides from decapod crustaceans. Three EsCFSHs from *E. sinensis* (marked in red) and homologous proteins from other decapod species (Table 2). The details of conserved cysteine residues and KR site were shown on the right side following the gene name.

By the comparison of amino acid sequences, the CFSHs of decapods could be categorized into three subtypes: type 1, type 2a, and type 2b (Figures 2, 3). The difference between type 1 and type 2 reflected on the existence of the signal peptide, while the difference between type 2a and type 2b reflected on the existence of the KR site. Additionally, the type 2b CFSHs contained two extra cysteine residues at the N-terminal and no N-glycosylation sites. The similarity between SpCFSH-1 and EsCFSH-1 was 68.9%, which was the highest among the compared CFSH sequences. Based on the sequence of 24 published CFSH mature peptides (Table 2), the phylogenetic tree confirmed that there were two main subtypes of CFSHs (Figure 4). In conclusion, EsCFSH-1 belonged to the type 1 CFSH.

### 3.3 The tissue expression of *EsCFSH-1* and *EsIAG* between the normal and intersex crabs

**FIGURE 5:**
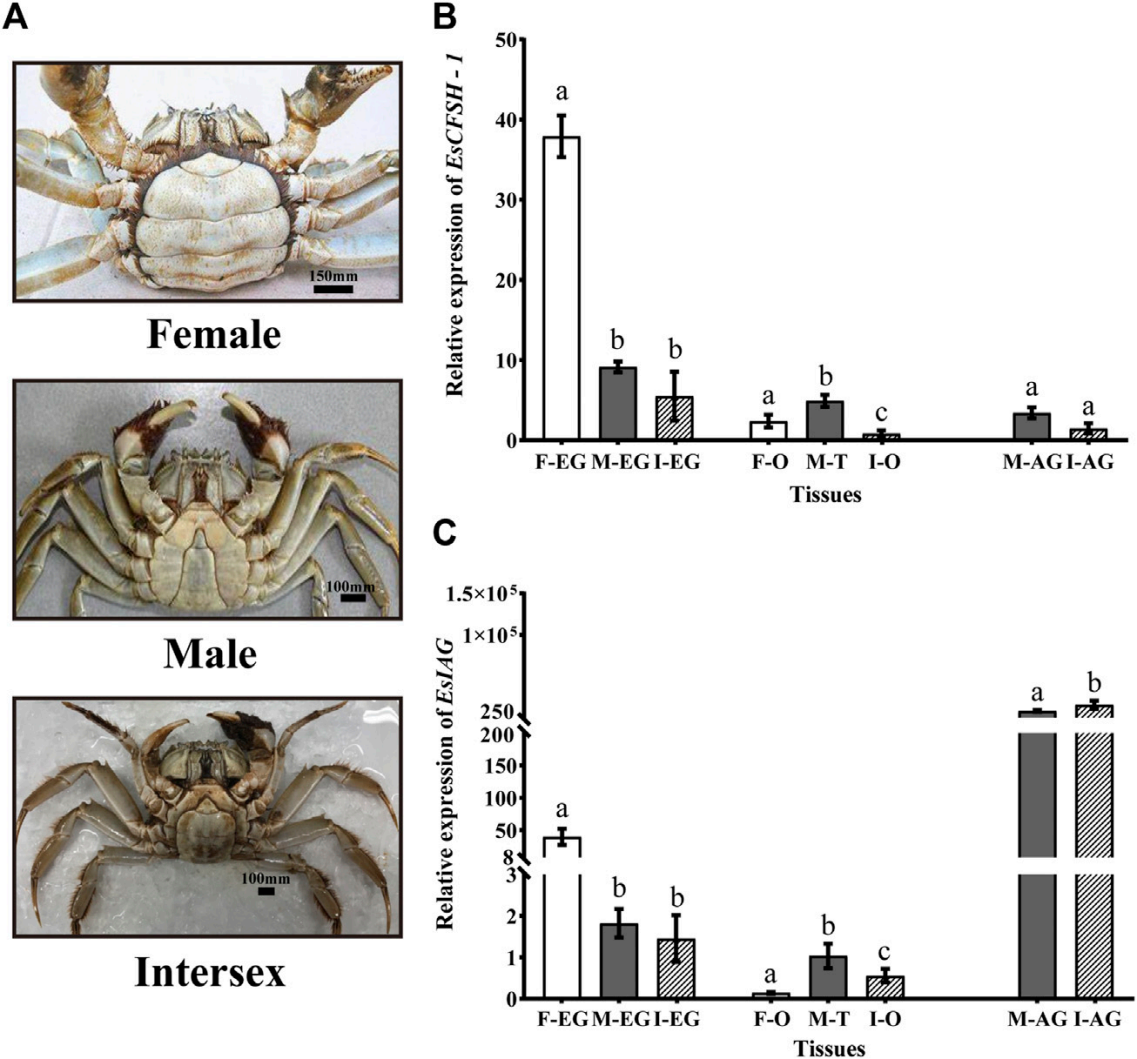
The distribution analysis of *EsCFSH-1* and *EsIAG* in the normal and intersex mitten crabs **(A)** The abdominal morphology of intersex crab was between normal female (round) and male (sharp) crabs **(B)** The comparative expression pattern of *EsCFSH-1* between the normal and intersex mitten crabs **(C)** Expression level of *EsIAG* in the normal and intersex mitten crabs. F-EG, eyestalk ganglion of females; M-EG, eyestalk ganglion of males; I-EG, eyestalk ganglion of intersexes; F-O, ovary of females; M-T, testis of males; I-O, ovary of intersexes; M-AG, androgenic gland of males; I-AG, androgenic gland of intersexes. The data were presented as means ± SEM of eight separate individuals (*n* = 8). Statistical significance was accepted at *p* < 0.05 and shown in different lower-case letters.

The abdomens of the intersex crabs exhibited an intermediate shape comparing to the narrow shape of male and the broad shape of female (Figure 5A). Each intersex crab possessed the female reproductive organ ovary and the male reproductive organ vas deferens attached by the androgenic gland despite it was genetically tested as female.

For *EsCFSH-1*, although it was expressed in AG and gonads, the main expression tissue was EG, and the expression in EG showed the higher level in the normal female crabs than the intersex crabs (*p* <0.05) and males (*p* <0.05) (Figure 5B).

Furthermore, for *EsIAG*, the expression in AG showed the higher level in the intersex crabs than the normal males (*p* <0.05) (Figure 5C). Besides, the EG of the normal females had the higher expression level than the males and even the intersex crabs (*p* <0.05) (Figure 5C).

### 3.4 The temporal comparison of *EsCFSH-1* and *EsIAG* expression in the embryos and larvae

**FIGURE 6:**
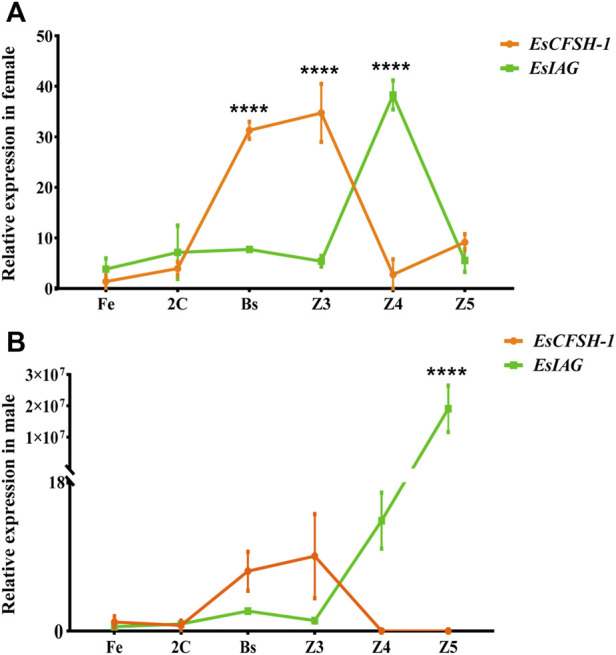
The expression pattern of *EsCFSH-1* and *EsIAG* in the female **(A)** and male **(B)** embryos and larvae. Fe, fertilized egg stage; 2C, two-cell stage; Bs, blastula stage; Z3, zoea III stage; Z4, zoea IV stage; Z5, zoea V stage. The data were presented as means ± SEM (*n* = 4). Significant differences of the gene expression levels were shown with four stars at *p* < 0. 0,001.

For the sex-distinguished embryo and larva (Figures 6A,B), the expression levels of *EsCFSH-1* and *EsIAG* both were low in the early stages of development (Fe and 2C), and then *EsCFSH-1* was higher expressed than *EsIAG* at the stages of Bs and Z3 (*p* <0.0001), while the expressed pattern of *EsCFSH-1* and *EsIAG* was reversed between the Z3 and Z4 stage. After the Z4 stage, the expression of *EsIAG* surged and *EsCFSH-1* declined sharply in male larva (*p* <0.0001), the trend was opposite in female larva.

### 3.5 The regulatory feedback loop between *EsCFSH-1* and *EsIAG*

**FIGURE 7:**
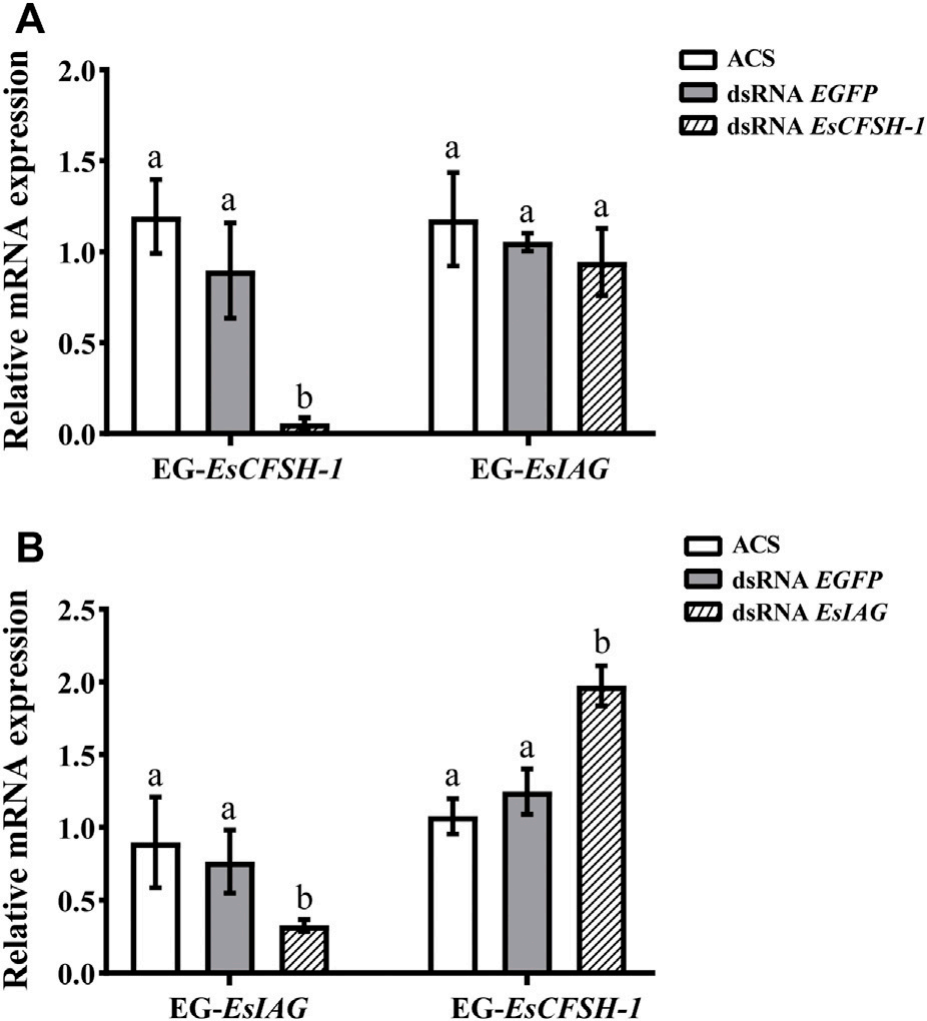
The expression relationships between *EsCFSH-1* and *EsIAG* after gene knockdown in the eyestalk ganglion of female *E. sinensis* **(A)** Silencing efficiency of *EsCFSH-1* and the expression changes of *EsIAG* **(B)** Silencing efficiency of *EsIAG* and the expression changes of *EsCFSH-1*. EG-*EsCFSH-1*, the expression of *EsCFSH-1* in the female EG; EG-*EsIAG*, the expression of *EsIAG* in the female EG. The data were presented as means ± SEM of four separate individuals (*n* = 4). Statistical significance was accepted at *p* < 0.05 and shown in different lower-case letters.

For the female crabs, the inhibition rate of *EsCFSH-1* expression was 89% after *EsCFSH-1* knockdown (*p* <0.05), and the expression of *EsIAG* in EG had no change (Figure 7A). Furthermore, the inhibition rate of *EsIAG* expression was 71% after *EsIAG* knockdown (*p* <0.05), and the expression of *EsCFSH-1* in EG rised significantly compared with the ACS and dsRNA *EGFP* groups (*p* <0.05) (Figure 7B).

**FIGURE 8:**
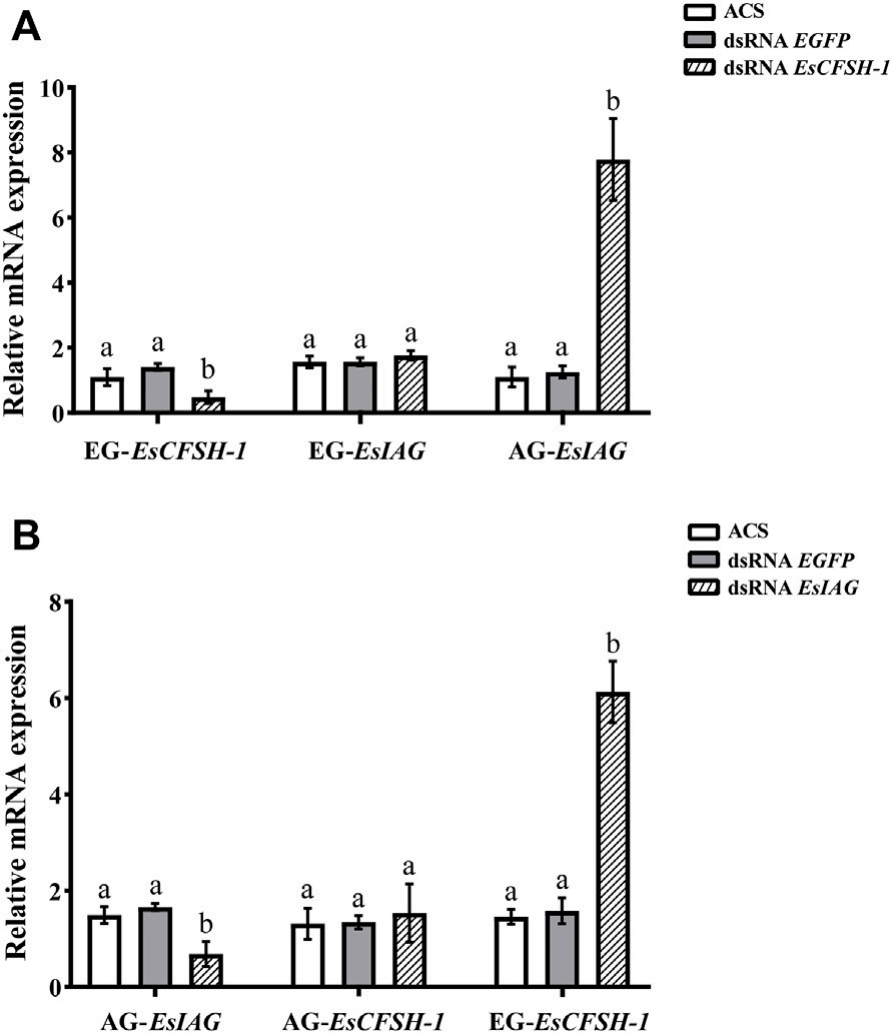
The expression relationships between *EsCFSH-1* and *EsIAG* after gene knockdown in the male *E. sinensis* **(A)** Silencing efficiency of *EsCFSH-1* and the expression changes of *EsIAG* **(B)** Silencing efficiency of *EsIAG* and the expression changes of *EsCFSH-1*. EG-*EsCFSH-1*, the expression of *EsCFSH-1* in the male EG; EG-*EsIAG*, the expression of *EsIAG* in the male EG; AG-*EsIAG*, the expression of *EsIAG* in the male AG; AG-*EsCFSH-1*, the expression of *EsCFSH-1* in the male AG. The data were presented as means ± SEM of four separate individuals (*n* = 4). Statistical significance was accepted at *p* <0.05 and shown in different lower-case letters.

For the male crabs, the expression of *EsCFSH-1* significantly decreased by 68% (*p* <0.05) after injecting with dsRNA *EsCFSH-1* in EG, and the expression of *EsIAG* in EG had no change, but a signifiant rising occurred in male AG (*p* <0.05) (Figure 8A). After the knockdown of *EsIAG*, the expression of *EsIAG* in AG decresed significantly by 62% (*p* <0.05), the expression of *EsCFSH-1* did not change in male AG, while it increased significantly in male EG (*p* <0.05) (Figure 8B).

To sum results up, there was a repression function from *EsCFSH-1* to *EsIAG* in female and male EGs, conversely, the inhibiting effect from *EsIAG* to *EsCFSH-1* only existed in male AG.

### 3.6 The impact of *EsCFSH-1* knockdown on the sexual phenotype

**FIGURE 9:**
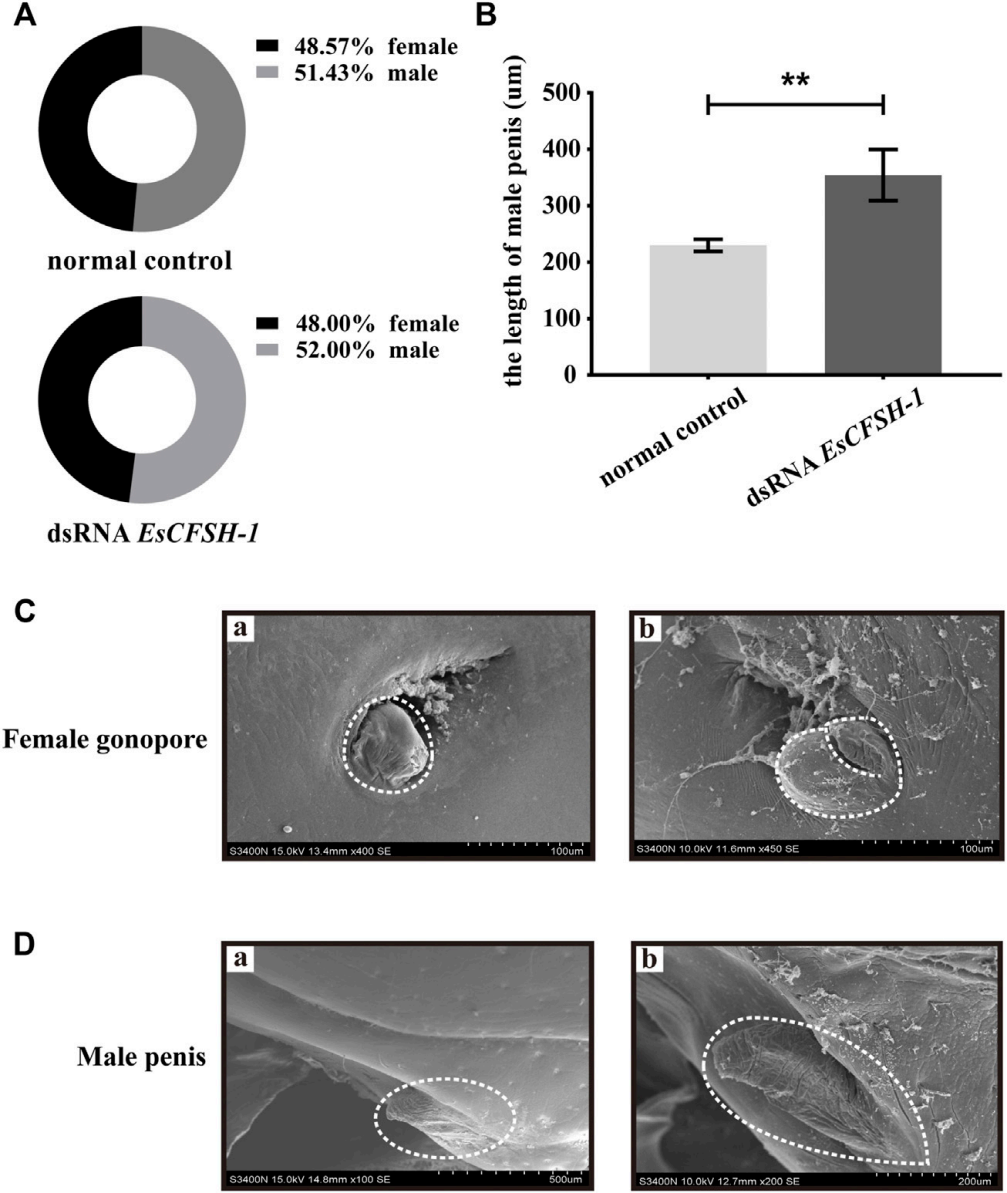
The female and male sexual characteristics of juvenile *E. sinensis* after *EsCFSH-1* knockdown **(A)** Sexual ratio of normal control group and dsRNA *EsCFSH-1* group **(B)** The length of male penis (μm). The data were presented as means ± SEM of four separate individuals (*n* = 3), and the double-stars indicated *p* <0.01 **(C)** The female gonopore characteristics before **(A)** and after **(B)** treated dsRNA *EsCFSH-1* **(D)** The male penis characteristics before **(A)** and after **(B)** treated dsRNA *EsCFSH-1*.

The knockdown of *EsCFSH-1* at the stage of juvenile I could not skew the sex ratio. For the crabs survived to the stage of juvenile III, there were 12 females and 13 males with a sex ratio of nearly 1:1 which was similar to the sex ratio of the control group (Figure 9A). However, the knockdown of *EsCFSH-1* appeared to have an impact on the development of external reproductive structures. The penis of a male juvenile became 140% larger after the treatment (Figures 9B,D), while the cover of female gonopore developed into an abnormal cleft structure in several cases (Figure 9C).

## 4 Discussion

EsCFSH-1 identified from genome of *E. sinensis* shared the typical protein structures of the type 1 CFSHs, including a signal peptide, a KR site, an IL-17 domain, and eight highly conserved cysteine residues. By comparison, the CFSH-2b subtype had the signal peptide but no KR site, while the CFSH-2a had the KR site but no signal peptide.

The signal peptide is necessary for the translocation of protein *via* the conventional secretory pathway (Rabouille, 2017). Therefore, CFSH-1 and 2b should belong to the secretory proteins, being transported to the plasma membrane through the endoplasmic reticulum and Golgi apparatus by COPII-coated vesicles (Palade, 1975; Brough et al., 2017). Based on the IL-17 domain, it is presumed that CFSH-1 participates in the cellular process of signal transduction. The protein with the IL-17 domain could mediate tyrosine phosphorylation to activate the JAK/STAT signaling pathway which transmits biochemical signals from IL-17 receptors to the nucleus of the regulated cell (Cooper and Adunyah, 1999). The KR site is important for the formation of mature protein of CFSH-1. It has been reported that the endocrine cells synthesize the peptide hormone by endoproteolysis of the precursor peptide at the site of Lys-Arg (KR) or Arg-Arg (RR) after the cleavage of the signal peptide (Hosaka et al., 1991; Veenstra, 2000).

Compared to the normal crabs, we found that the intersex crabs show the aberrant expression of *EsCFSH-1* and *EsIAG*. Normally, *CFSH-1* expressed at the highest level in the female EG (Kotaka and Ohira, 2017; Liu et al., 2018; Liu et al., 2021a) and *IAG* extremely highly expressed in the male specific organ AG (Sagi and Aflalo, 2005). For the intersex crabs, the expression of *EsCFSH-1* and *EsIAG* in the EG and AG was close to the male level. Given these intersex crabs are genetic females, the masculinized phenotype may be related to the lowered *EsCFSH-1* expression and the increased *EsIAG* expression, which eventually lead to the unorthodox emergence of AG. Furthermore, as the demethylation of 5′-UTR region of *CFSH* ensured the high expression of *CFSH* in the female EG (Jiang et al., 2020a), our ongoing study will find out whether an abnormally high methylation of this region decrease the expression of *EsCFSH-1* in the intersex crabs.

Next, we examined the role of *EsCFSH-1* and *EsIAG* in the process of sexual sex differentiation. Based on our results, it seems that *EsCFSH-1* initiates feminization by an effective repression of *EsIAG* expression since the late stage of embryogenesis. In the male perspective, the early repression of *EsIAG* appears inefficient, which results in the surge of *EsIAG* expression during the transition after Z4 to megalopa. In a word, the change trends of the two genes were almost same before the Z4 stage, at which could be confirmed as the key period when the feedback loop relationship between *EsCFSH-1* and *EsIAG* worked. Our finding is consistent with the morphological observation of *Eriocheir japonicus*, in which the sexual differentiation of internal reproductive organ (gonoduct) could not be observed until the megalopa stage (Lee et al., 1994).

The experiment of gene knockdown was later conducted to figure out the regulatory relationship between *EsCFSH-1* and *EsIAG*. Our results confirmed the inhibitory effect of *EsCFSH-1* to *EsIAG* in the AG, which agree with the finding in the mud crab *S. paramamosain* (Liu et al., 2018). We also found the inhibitory effect of *EsIAG* to *EsCFSH-1* in the EG of male and female, which is supported by the finding in the peppermint shrimp *L. vittate* (Liu et al., 2021a; Liu et al., 2021c). Our study confirmed the existence of the regulatory feedback loop between *EsCFSH-1* and *EsIAG*. In *S. paramamosain*, *CFSH* negatively regulated *IAG* by inhibiting *STAT* (signal transducers and activators of transcription), which was a key transcription factor of *IAG* (Jiang et al., 2020c). Further studies are required to verify the inhibitory mechanism of *CFSH* to *IAG* in male *E. sinensis* and more importantly investigate the inhibitory mechanism of *IAG* to *CFSH*.

Our study also revealed that *EsCFSH-1* regulates the development of reproductive system during the sexual sex differentiation. The disruption of *EsCFSH-1* expression could lead to the deformed cover of female gonopores and the elongation of male penises of juveniles. The impaired development of female gonopores was also observed in *C. sapidus* (Zmora and Chung, 2014) and *S. paramamosain* (Jiang et al., 2020b) after the *CFSH* knockdown in juvenile females. As the positive correlation has been well established between the *IAG* expression and the development of male sex characteristics (Khalaila et al., 2001), the over-growth of penis should be explained by the compromised inhibitory effect of *EsCFSH-1* to *EsIAG*.

In summary, we compared the gene expression of *EsCFSH-1* and *EsIAG* between normal and intersex crabs, and between sex-distinguished embryos and larvae at various developmental stages. Next, we confirmed the existence of the regulatory feedback loop between *EsCFSH-1* and *EsIAG*. At last but not least, we demonstrated the relationship between *EsCFSH-1* and the development of reproductive system. Our study provides strong evidence for the feminization function of *CFSH-1*, which contributes to a better understanding of the molecular mechanism of sexual sex differentiation.

## Data Availability

The datasets presented in this study can be found in online repositories. The names of therepository/repositories and accession number(s) can be found below: https://www.ncbi.nlm.nih.gov/genbank/, OP351640.

## References

[B1] Adkins-Regan E. (2014). A new hormone negates a default principle. Endocrinology 155, 10–11. 10.1210/en.2013-2078 24364585

[B2] Almagro Armenteros J. J. Tsirigos K. D. Sonderby C. K. Petersen T. N. Winther O. Brunak S. (2019). SignalP 5.0 improves signal peptide predictions using deep neural networks. Nat. Biotechnol. 37, 420–423. 10.1038/s41587-019-0036-z 30778233

[B3] Bairoch A. Boeckmann B. (1991). The SWISS-PROT protein sequence data bank. Nucleic Acids Res. 19, 2247–2249. 10.1093/nar/19.suppl.2247 2041811 PMC331359

[B4] Brough D. Pelegrin P. Nickel W. (2017). An emerging case for membrane pore formation as a common mechanism for the unconventional secretion of FGF2 and IL-1β. J. Cell. Sci. 130, 3197–3202. 10.1242/jcs.204206 28871048

[B5] Cooper S. V. S. R. S. Adunyah S. E. (1999). Evidence for the involvement of JAK STAT pathway in the signaling mechanism of interleukin-17. Biochem. Biophys. Res. Commun. 262, 14–19. 10.1006/bbrc.1999.1156 10448060

[B6] Cui Z. Du J. Yang Y. Liu Y. (2018). A specific molecular marker for identifying the sex of Eriocheir sinensis. China Pat. Appl. 4, 201811449018.

[B7] Cui Z. Liu Y. Yuan J. Zhang X. Ventura T. Ma K. Y. (2021). The Chinese mitten crab genome provides insights into adaptive plasticity and developmental regulation. Nat. Commun. 12, 2395. 10.1038/s41467-021-22604-3 33888695 PMC8062507

[B8] Farhadi A. Cui W. Zheng H. Li S. Zhang Y. Ikhwanuddin M. (2021). The regulatory mechanism of sexual development in decapod Crustaceans. Front. Mar. Sci. 8, 679687. 10.3389/fmars.2021.679687

[B9] Ferre F. Clote P. (2005). DiANNA: A web server for disulfide connectivity prediction. Nucleic Acids Res. 33, 230–232. 10.1093/nar/gki412 PMC1160173 15980459

[B10] Fu C. Li F. Wang L. Wu F. Wang J. Fan X. (2020). Molecular characteristics and abundance of insulin-like androgenic gland hormone and effects of RNA interference in *Eriocheir sinensis* . Anim. Reprod. Sci. 215, 106332. 10.1016/j.anireprosci.2020.106332 32216938

[B11] Gupta R. Brunak S. (2002). Prediction of glycosylation across the human proteome and the correlation to protein function. Pac. Symp. Biocomput. 7, 310–322. 11928486

[B12] Hosaka M. Nagahama M. Kim W. S. Watanabe T. Hatsuzawa K. Ikemizu J. (1991). Arg-X-Lys/Arg-Arg motif as a signal for precursor cleavage catalyzed by furin within the constitutive secretory pathway. J. Biol. Chem. 266, 12127–12130. 10.1016/s0021-9258(18)98867-8 1905715

[B13] Jiang Q. Lin D. Huang H. Wang G. Ye H. (2020a). DNA methylation inhibits the expression of CFSH in mud crab. Front. Endocrinol. 11, 163. 10.3389/fendo.2020.00163 PMC7160318 32328029

[B14] Jiang Q. Lu B. Lin D. Huang H. Chen X. Ye H. (2020b). Role of crustacean female sex hormone (CFSH) in sex differentiation in early juvenile mud crabs, *Scylla paramamosain* . Gen. Comp. Endocrinol. 289, 113383. 10.1016/j.ygcen.2019.113383 31904358

[B15] Jiang Q. Lu B. Wang G. Ye H. (2020c). Transcriptional inhibition of sp-IAG by Crustacean female sex hormone in the mud crab, *Scylla paramamosain* . Int. J. Mol. Sci. 21, 5300. 10.3390/ijms21155300 PMC7432471 32722594

[B16] Jones D. T. (1999). Protein secondary structure prediction based on position-specific scoring matrices. J. Mol. Biol. 292, 195–202. 10.1006/jmbi.1999.3091 10493868

[B17] Khalaila I. Katz T. Abdu U. Yehezkel G. Sagi A. Sagi A. (2001). Effects of implantation of hypertrophied androgenic glands on sexual characters and physiology of the reproductive system in the female red claw crayfish, *Cherax quadricarinatus* . Gen. Comp. Endocrinol. 121, 242–249. 10.1006/gcen.2001.7607 11254366

[B18] Kotaka S. Ohira T. (2017). cDNA cloning and *in situ* localization of a crustacean female sex hormone-like molecule in the kuruma prawn *Marsupenaeus japonicus* . Fish. Sci. 84, 53–60. 10.1007/s12562-017-1152-7

[B19] Lee T.-H. Yamauchi M. Yamazaki F. (1994). Sex differentiation in the crab *Eriocheir japonicus* (Decapoda, Grapsidae). Invertebr. Reproduction Dev. 25, 123–137. 10.1080/07924259.1994.9672377

[B20] Letunic I. Bork P. (2018). 20 years of the SMART protein domain annotation resource. Nucleic Acids Res. 46, D493–D496. 10.1093/nar/gkx922 29040681 PMC5753352

[B21] Levy T. Rosen O. Simons O. Alkalay A. S. Sagi A. (2017). The gene encoding the insulin-like androgenic gland hormone in an all-female parthenogenetic crayfish. PLoS ONE 12, e0189982. 10.1371/journal.pone.0189982 29261765 PMC5738133

[B22] Levy T. Sagi A. (2020). The "IAG-Switch"-A key controlling element in decapod Crustacean sex differentiation. Front. Endocrinol. 11, 651. 10.3389/fendo.2020.00651 PMC7511715 33013714

[B23] Levy T. Tamone S. L. Manor R. Aflalo E. D. Sklarz M. Y. Chalifa-Caspi V. (2020). The IAG-switch and further transcriptomic insights into sexual differentiation of a protandric shrimp. Front. Mar. Sci. 7, 587454. 10.3389/fmars.2020.587454

[B24] Liang X. Yan S. Zheng C. Guo D. (1974). The larval development of *Eriocheir sinensis* H. milne Edwards. ACTA ZOOL. SIN. 20, 61–75.

[B25] Liu A. Liu J. Liu F. Huang Y. Wang G. Ye H. (2018). Crustacean female sex hormone from the mud crab *Scylla paramamosain* is highly expressed in prepubertal males and inhibits the development of androgenic gland. Front. Physiol. 9, 924. 10.3389/fphys.2018.00924 30065661 PMC6056722

[B26] Liu F. Shi W. Huang L. Wang G. Zhu Z. Ye H. (2021a). Roles of Crustacean female sex hormone 1a in a protandric simultaneous hermaphrodite shrimp. Front. Mar. Sci. 8, 791965. 10.3389/fmars.2021.791965

[B27] Liu F. Shi W. Ye H. Liu A. Zhu Z. (2021b). RNAi reveals role of insulin-like androgenic gland hormone 2 (IAG2) in sexual differentiation and growth in hermaphrodite shrimp. Front. Mar. Sci. 8, 666763. 10.3389/fmars.2021.666763

[B28] Liu F. Shi W. Ye H. Zeng C. Zhu Z. (2021c). Insulin-like androgenic gland hormone 1 (IAG1) regulates sexual differentiation in a hermaphrodite shrimp through feedback to neuroendocrine factors. Gen. Comp. Endocrinol. 303, 113706. 10.1016/j.ygcen.2020.113706 33359802

[B29] Livak K. J. Schmittgen T. D. (2001). Analysis of relative gene expression data using real-time quantitative PCR and the 2^−ΔΔCt^ Method. METHODS 25, 402–408. 10.1006/meth.2001.1262 11846609

[B30] Misener S. Krawetz S. A. (2000). Bioinformatics methods and protocols. Totowa, NJ: Humana.

[B31] Montú M. Anger K. De Bakker C. (1996). Larval development of the Chinese mitten crab *Eriocheir sinensis* H. Milne-edwards (Decapoda: Grapsidae) reared in the laboratory. Helgol. Meeresunters. 50, 223–252. 10.1007/BF02367153

[B32] Palade G. (1975). Intracellular aspects of the process of protein synthesis. Science 189, 347–358. 10.1126/science.1096303 1096303

[B33] Priyadarshi H. Das R. Pavan-Kumar A. Gireesh-Babu P. Javed H. Kumar S. (2017). Silencing and augmentation of IAG hormone transcripts in adult *Macrobrachium rosenbergii* males affects morphotype transformation. J. Exp. Biol. 220, 4101–4108. 10.1242/jeb.163410 28851817

[B34] Rabouille C. (2017). Pathways of unconventional protein secretion. Trends Cell. Biol. 27, 230–240. 10.1016/j.tcb.2016.11.007 27989656

[B35] Sagi A. Aflalo E. D. (2005). The androgenic gland and monosex culture of freshwater prawn *Macrobrachium rosenbergii* (de man): A biotechnological perspective. Aquac. Res. 36, 231–237. 10.1111/j.1365-2109.2005.01238.x

[B36] Saitou N. Nei M. (1987). The neighbor-joining method a new method for reconstructing phylogenetic trees. Mol. Biol. Evol. 4, 406–425. 10.1093/oxfordjournals.molbev.a040454 3447015

[B37] Song C. Liu L. Hui M. Liu Y. Liu H. Cui Z. (2018). Primary molecular basis of androgenic gland endocrine sex regulation revealed by transcriptome analysis in *Eriocheir sinensis* . J. Oceanol. Limnol. 37, 223–234. 10.1007/s00343-019-7254-6

[B38] Steentoft C. Vakhrushev S. Y. Joshi H. J. Kong Y. Vester-Christensen M. B. Schjoldager K. T. (2013). Precision mapping of the human O-GalNAc glycoproteome through Simple Cell technology. EMBO J. 32, 1478–1488. 10.1038/emboj.2013.79 23584533 PMC3655468

[B39] Stoline M. R. (1981). The status of multiple comparisons simultaneous estimation of all pairwise comparisons in one-way ANOVA designs. Am. Statistician 35, 134–141. 10.1080/00031305.1981.10479331

[B40] Swift M. L. (1997). GraphPad prism, data analysis, and scientific graphing. J. Chem. Inf. Comput. Sci. 37, 411–412. 10.1021/ci960402j

[B41] Toyota K. Miyakawa H. Hiruta C. Sato T. Katayama H. Ohira T. (2021). Sex determination and differentiation in decapod and cladoceran Crustaceans: An overview of endocrine regulation. Genes. 12, 305. 10.3390/genes12020305 33669984 PMC7924870

[B42] Tsutsui N. Kotaka S. Ohira T. Sakamoto T. (2018). Characterization of distinct ovarian isoform of crustacean female sex hormone in the kuruma prawn *Marsupenaeus japonicus* . Comp. Biochem. Physiol. A Mol. Integr. Physiol. 217, 7–16. 10.1016/j.cbpa.2017.12.009 29277431

[B43] Veenstra J. A. (2000). Mono- and dibasic proteolytic cleavage sites in insect neuroendocrine peptide precursors. Archives Insect Biochem. Physiology 43, 49–63. 10.1002/(SICI)1520-6327(200002)43:2<49::AID-ARCH1>3.0.CO;2-M 10644969

[B44] Ventura T. Manor R. Aflalo E. D. Weil S. Rosen O. Sagi A. (2012). Timing sexual differentiation: Full functional sex reversal achieved through silencing of a single insulin-like gene in the prawn, *Macrobrachium rosenbergii* . Biol. Reprod. 86, 90–96. 10.1095/biolreprod.111.097261 22133694

[B45] Ye J. Coulouris G. Zaretskaya I. Cutcutache I. Rozen S. Madden T. L. (2012). Primer-BLAST: A tool to design target-specific primers for polymerase chain reaction. BMC Bioinforma. 13, 134. 10.1186/1471-2105-13-134 PMC3412702 22708584

[B46] Zmora N. Chung J. S. (2014). A novel hormone is required for the development of reproductive phenotypes in adult female crabs. Endocrinology 155, 230–239. 10.1210/en.2013-1603 24280057

